# Does ultrasonic activation improve the bond strength and root canal filling quality of endodontic sealers?

**DOI:** 10.1590/0103-6440202204728

**Published:** 2022-06-24

**Authors:** Karine Padoin, Thais Camponogara Bohrer, Lucas Galle Ceolin, Carlos Alexandre Souza Bier, Ricardo Abreu da Rosa, Renata Dornelles Morgental

**Affiliations:** 1 Graduate Program in Dental Sciences, Federal University of Santa Maria (UFSM), Santa Maria, RS, Brazil.; 2 School of Dentistry, Federal University of Santa Maria(UFSM), Santa Maria, RS, Brazil.; 3 Graduate Program in Dentistry, Federal University of Rio Grande do Sul (UFRGS), Porto Alegre, RS, Brazil.

**Keywords:** endodontics, root canal filling materials, root canal obturation, ultrasonic

## Abstract

This study aimed to investigate the effect of ultrasonic activation (UA) of three endodontic sealers on the bond strength to root dentin and root canal filling quality. Ninety six bovine incisors were instrumented and root canal filling was carried out using AH Plus (AP), Sealer Plus (SP), or Sealer Plus BC (BC), with or without UA (n=16/group). Two 1.5-mm slices were obtained from each root third. The first slice was subjected to push-out testing and failure mode analysis, while the second was observed under a stereomicroscope for filling quality assessment. Data were analyzed by Kruskal-Wallis, Mann-Whitney and Friedman tests (α=0.05). SP showed higher bond strength and fewer voids than BC in the apical third and when root thirds data were pooled. SP also had higher bond strength compared with AH Plus in the apical third. UA improved the bond strength when BC was used but did not affect the filling quality of any sealer. There were no significant differences between the ultrasonically activated sealers regarding bond strength and filling quality. When root thirds were compared, the bond strength was similar along the root, but there was a tendency to worsen filling quality, with more voids, in the apical segment. In conclusion, UA was effective in increasing the bond strength of the calcium silicate-based sealer but did not improve its filling quality. For the epoxy resin-based sealers, these properties were not affected by UA.

## Resumo

Este estudo teve como objetivo investigar o efeito da ativação ultrassônica (AU) de três cimentos endodônticos na resistência de união à dentina radicular e na qualidade da obturação do canal radicular. Noventa e seis incisivos bovinos foram instrumentados e a obturação dos canais radiculares foi realizada com AH Plus (AP), Sealer Plus (SP) ou Sealer Plus BC (BC), com ou sem AU (n=16/grupo). Duas fatias de 1,5 mm foram obtidas de cada terço radicular. A primeira fatia foi submetida ao teste push-out e análise de modo de falha, enquanto a segunda foi observada em um estereomicroscópio para avaliação da qualidade da obturação. Os dados foram analisados ​​por testes de Kruskal-Wallis, Mann-Whitney e Friedman (α=0,05). SP mostrou maior resistência de união e menos espaços vazios na massa obturadora do que BC no terço apical e quando os dados dos terços radiculares foram agrupados. SP também apresentou maior resistência de união em comparação ao AH Plus no terço apical. A AU melhorou a resistência de união quando BC foi usado, mas não afetou a qualidade da obturação de nenhum dos cimentos. Não houve diferença significante entre os cimentos ativados por ultrassom em relação à resistência de união e qualidade da obturação. Quando comparados os terços radiculares, a resistência de união foi semelhante ao longo da raiz, mas houve uma tendência de pior qualidade no preenchimento, com mais vazios, no terço apical. Concluindo, a AU foi eficaz em aumentar a resistência de união do cimento à base de silicato de cálcio, mas não melhorou a qualidade da obturação. Para os cimentos à base de resina epóxi, essas propriedades não foram afetadas pela AU.

## Introduction

Ultrasound was first introduced to Endodontics in the 1950s and has been used in several endodontic procedures, ranging from access cavity refinement to apical surgery ^1^. Currently, it is mainly used for the agitation of irrigating solutions, without simultaneous ultrasonic instrumentation, the so-called passive ultrasonic irrigation (PUI) ^2^. It promotes acoustic streaming and cavitation forces that lead to greater removal of organic and inorganic debris from the root canal system ^1^. PUI promotes better cleaning of the dentinal walls ^3^ and, therefore, may improve the antimicrobial action of intracanal dressings ^2^ and the penetration of endodontic sealers into the dentinal tubules ^4^.

The application of ultrasonic devices in obturation procedures has also been proposed, aiming to improve root canal filling quality ^5^ ^,^ ^6^. Previous studies have shown that the ultrasonic activation of endodontic sealers may promote: higher bond strength to root dentin ^4^ ^,^ ^7^, greater penetration of sealers into isthmuses ^8^, and dentinal tubules ^4^ ^,^ ^7^, in addition to better interfacial adaptation between the filling material and the root canal walls ^5^ ^,^ ^8^.

Nevertheless, the chemical composition and viscosity of endodontic sealers vary greatly, which may interfere with ultrasonic activation ^7^, producing different results. AH Plus (Dentsply DeTrey Gmbh, Konstanz, Germany), an epoxy resin-based sealer, has been extensively investigated, and it is considered the ‘gold-standard’ among endodontic sealers due to its excellent physicochemical properties ^9^, biological behavior ^10^, and adhesion to dentin ^7^.

New endodontic sealers have been introduced to the market, such as Sealer Plus (MK Life, Porto Alegre, RS, Brazil). It is another epoxy resin-based sealer and, compared to AH Plus, it showed similar solubility, flow, and pH, but lower radiopacity and setting time ^11^. Yet, both sealers presented physicochemical properties in accordance with ISO 6876:2012 recommendations. Unlike AH Plus, Sealer Plus contains calcium hydroxide, which may explain its low cytotoxicity and high biocompatibility ^12^.

Calcium silicate-based endodontic sealers have received considerable attention from the scientific community in the last years due to their biological properties and potential for biomineralization ^9^. A new pre-mixed calcium silicate-based sealer, Sealer Plus BC (MK Life, Porto Alegre, RS, Brazil), is commercially available. In recent investigations, the material displayed good biocompatibility, inducing mild or absent inflammation at 30 days ^13^. Regarding its physicochemical characteristics, Sealer Plus BC presented greater solubility and volumetric change than AH Plus ^14^. There is scarce information about the behavior of these new endodontic sealers under ultrasonic activation ^4^.

In this context, this study aimed to evaluate the effect of ultrasonic activation of two epoxy resin-based sealers (AH Plus and Sealer Plus) and one bioceramic sealer (Sealer Plus BC) on their bond strength to root dentin and root canal filling quality. The null hypothesis tested was that the ultrasonic activation of sealers would not influence bond strength and filling quality.

## Material and methods

### Sample size calculation

The sample size was calculated using the parameters described by Wiesse et al. ^7^: bond strength of 1.65 (( 0.45) MPa in the non-activated sealer (AH Plus) and 2.25 (( 0.56) MPa in the ultrasonically activated sealer; 90% power; 5% significance level (www.openepi.com/SampleSize/SSMean.htm). The estimated minimum sample size was found to be 16 per group.

### Sample selection and preparation

Ninety six bovine mandibular incisors from animals killed for commercial reasons were used. Teeth were obtained immediately after extraction and kept in 0.9% saline solution at 4ºC until the following methodological steps. The external surfaces were cleaned with periodontal curettes (Golgran, São Paulo, SP, Brazil) and teeth were disinfected by immersion in 0.5% chloramine-T solution (Sigma-Aldrich, St. Louis, MO, USA) at 4ºC for a week. Specimens were observed under a digital stereomicroscope (Stereo Discovery V20; Zeiss, Oberkochen, Germany) at 8× magnification, and those with cracks, incomplete root formation, apical opening larger than a size 70 K-file (Dentsply-Maillefer, Ballaigues, Switzerland), or other structural anomalies were excluded.

Teeth were decoronated with carborundum discs (KG Sorensen, Barueri, SP, Brazil) to standardize a remaining root length of 20 mm. Initially, root canals were irrigated with 5 mL of 2.5% sodium hypochlorite solution (NaOCl; Biodinâmica, Ibiporã, PR, Brazil). A size 15 K-file (Dentsply-Maillefer, Ballaigues, Switzerland) was passively introduced into the root canal until its tip was visible at the apical foramen. This procedure was also performed under the digital stereomicroscope at 8× magnification. The working length (WL) was determined 1 mm shorter of this landmark. Root canal preparation was carried out by a crown-down technique, using size 5 and 4 Gates-Glidden drills (Dentsply-Maillefer, Ballaigues, Switzerland) up to the coronal (6 mm) and middle (12 mm) root thirds, respectively. The apical third was prepared by hand stainless steel instruments, from a size 70 to 100 K-file (Dentsply-Maillefer, Ballaigues, Switzerland).

All procedures were performed under copious irrigation, using 2 mL of 2.5% NaOCl at each instrument change. After chemomechanical preparation, root canals were irrigated with 5 mL of 17% EDTA (Biodinâmica, Ibiporã, PR, Brazil) for 5 minutes to remove the smear layer, followed by 10 mL of distilled water ^8^. Root canals were dried with absorbent paper points (Dentsply Brazil, Petrópolis, RJ, Brazil). Size 100/.02 taper gutta-percha points (Dentsply Brazil, Petrópolis, RJ, Brazil) were tested for tug-back at the WL, and the apical position was confirmed radiographically.

### Root canal filling

Specimens were randomly distributed into six experimental groups, using www.randomization.com, according to the endodontic sealer (Table 1) and type of sealer activation:

- AP: AH Plus without activation;
- APU: AH Plus with ultrasonic activation;
- SP: Sealer Plus without activation;
- SPU: Sealer Plus with ultrasonic activation;
- BC: Sealer Plus BC without activation;
- BCU: Sealer Plus BC with ultrasonic activation.

**Table 1:** Endodontic sealers tested and their compositions.

Sealer	Composition	Manufacturer
*AH Plus*	Paste A: bisphenol-A epoxy resin; bisphenol-F epoxy resin; calcium tungstate; zirconium oxide; silica and iron oxide.	Dentsply, DeTrey GmbH, Konstanz, Germany
	Paste B: adamantine amine; n, n "-dibenzyl-5-oxanone diamine-1,9; TCD-diamine; calcium tungstate; zirconium oxide; silica and silicone oil.	
*Sealer Plus*	Base paste: bisphenol-A-coepichlorohydrin; bisphenol-F epoxy resin; zirconium oxide; silicon and siloxanes; iron oxide; calcium hydroxide.	MK Life, Porto Alegre, RS, Brazil
	Catalytic paste: hexamethylenetetramine; zirconium oxide; silicon and siloxanes; calcium hydroxide; tungstate calcium.	
*Sealer Plus BC*	Zirconium oxide; tri-calcium silicate; di-calcium silicate; calcium hydroxide; propylene glycol.	MK Life, Porto Alegre, RS, Brazil

Sealers were manipulated according to the manufacturer's instructions and inserted into the canals with a caliber 40 Lentulo spiral (Dentsply-Maillefer, Ballaigues, Switzerland) at low speed for 5 seconds. This procedure was repeated (up to three times) until the root canal walls were completely covered by the sealer. In the groups of ultrasonically activated sealers (APU, SPU, and BCU), activation was performed immediately after sealer placement, using an E1 Irrisonic tip (Helse Ultrasonic, Ribeirão Preto, SP, Brazil) attached to an ultrasonic device (Sonic Laxis BP LED, Schuster, Santa Maria, RS, Brazil), 2 mm short of the WL, at 20% power level. As the ultrasonic insert oscillates in a single plane, it was activated for 20 seconds in the mesiodistal direction and another 20 seconds in the buccolingual direction ^8^. Gentle brushing movements were performed against the root canal walls.

Next, in all groups, a size 100/.02 taper gutta-percha point was inserted into the full WL and the root canal obturation was complemented by the lateral condensation technique with a D-size finger spreader (Dentsply-Maillefer, Ballaigues, Switzerland), inserted up to 2 mm shorter of the WL, and size FM accessory gutta-percha points (Dentsply-Maillefer, Ballaigues, Switzerland). After radiographic confirmation of complete root canal filling, the excess of material was removed by a heated plugger (Golgran, São Paulo, SP, Brazil) 2 mm below the canal orifice, then cold vertical compaction was performed. The specimens were sealed with a temporary restorative material (Coltosol; Coltene, Altstätten, Switzerland) and stored at 37ºC and 100% humidity for 24 hours to allow the sealers to set ^7^.

Specimens were transversally sectioned using a precision cutting machine (Isomet; Extec Corp, Enfield, CT, USA) set at 300 rpm and equipped with a double-sided diamond disc (Buehler, Lake Bluff, IL, USA). Their coronal portion (4 mm) was included in self-cured acrylic resin (Clássico, Campo Lindo Paulista, SP, Brazil) to facilitate fixation to the machine. The most coronal and apical parts (2 mm) of each specimen were discarded, and six 1.5 mm-thick (± 0.3 mm) slices were produced from each root, two per root third (coronal, middle, and apical). The first slice (the most coronal) was subjected to push-out testing and failure mode analysis, while the second was used for filling quality assessment.

### Push-out bond strength test

The push-out test was performed in a universal testing machine (EMIC DL-2000; EMIC, São José dos Pinhais, PR, Brazil). Root slices were positioned in the machine with their coronal surfaces facing down in a metal device with an opening of approximately 4 mm in diameter. The root canal orifice of each slice was centered in this opening. The slices were then submitted to compression loading using a metallic plunger with a 0.8 mm-diameter tip touching the root canal filling center. Loading forces were introduced from an apical to a coronal direction (1 mm/min speed) (15, 16), and the bond strength (σ) was obtained in megapascal (MPa). The following formula was applied: σ = F/A, where F = load for filling dislodgement (N) and A = adhesion area (mm^2^), as previously described ^15^. To determine A, the following formula was used: A = πg (R_1_ + R_2_), where π = 3.14, g = slant height, R_1_ = smaller base radius, R_2_ = larger base radius. To determine g, the following calculation was used: g^2^= (H^2^ + [R_1_ - R_2_]^2^), where H = section height. R_1_ and R_2_ were obtained by measuring the internal diameters of the smallest and largest base, respectively, corresponding to the inner diameters of the root canal walls. H, R_1_, and R_2_ were measured with a digital caliper before the push-out test (Mitutoyo, Suzano, SP, Brazil).

### Failure mode analysis

After the push-out test, slices were analyzed by a blinded and calibrated (kappa=0.83) examiner using a digital stereomicroscope (Stereo Discovery V20; Zeiss, Oberkochen, Germany) at 25× magnification to determine the failure pattern, as described previously ^17^. The examiner was trained by an experienced endodontic professor (inter-examiner kappa=0.75). Failures were classified as adhesive when the sealer was completely separated from dentin (surface without sealer), cohesive when the failure occurred within the filling material (dentin surface entirely covered by sealer), and mixed when a mixture of adhesive and cohesive modes occurred (dentin surface partially covered by sealer).

### Filling quality assessment

The second slices obtained from each root third were used to investigate the filling quality promoted by the different sealers and types of activation. They were observed under a digital stereomicroscope (Stereo Discovery V20; Zeiss, Oberkochen, Germany) at 25× magnification. Digital images were obtained and evaluated to estimate the presence, number, and diameter of voids within the filling material, using a four-point scoring system, adapted from Kim et al. ^6^. For void diameter calculation, the ImageJ 1.46 software (National Institutes of Health, Bethesda, MD, USA) was used with a standardized 75% zoom.

Filling quality was assessed by a blinded and calibrated (weighted kappa=0.84) examiner, who was previously trained by an experienced endodontic professor (inter-examiner weighted kappa=0.79). The following scores were considered: 1) well-condensed filling material with only a few voids (< 0.1 mm in diameter); 2) imperfectly condensed filling with some small voids (more than 3 defects) or medium-sized voids (0.1 to 0.2 mm in diameter); 3) inadequately condensed filling with several small voids (more than 5 defects) or large voids (> 0.2 mm in diameter); 4) poorly condensed filling, presenting many small voids (more than 7 defects) or void space connecting separate root canal walls ^6^.

### Statistical analysis

Bond strength data were submitted to the Shapiro-Wilk test and showed non-normal distribution. The Kruskal-Wallis test was used to compare sealers and the Mann-Whitney test to compare types of activation. Friedman tests were applied for repeated measures in the same group, i.e. comparison between root thirds. Void scores were analyzed similarly. The significance level was set at 5% (SPSS Statistics 20 software; IBM SPSS Inc., Chicago, IL, USA).

## Results

### Push-out bond strength

Bond strength results are summarized in Table 2. For non-ultrasonically activated sealers, SP had higher bond strength than BC in the apical third and the overall analysis, i.e. when root thirds data were pooled (P=0.001). SP also showed higher bond strength values than AP in the apical third (P=0.021). There was no significant difference between ultrasonically activated sealers in any root segment (P>0.05).

Ultrasonic activation of BC resulted in higher bond strength than no activation in the apical third (P=0.042) and overall (P=0.011). The ultrasound did not affect the bond strength of AP and SP. No significant difference was detected between root thirds for any sealer, regardless of ultrasonic activation (P>0.05).

### Failure mode

**Figure 1:**
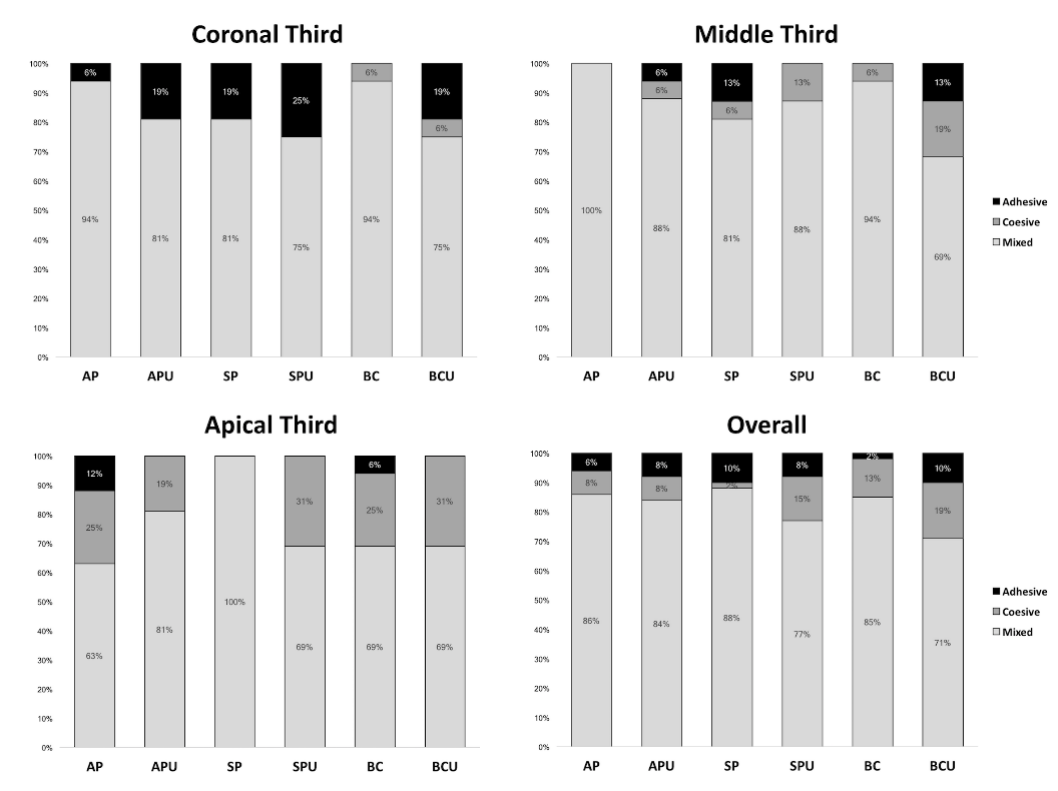
Failure mode distribution (%) according to sealer, type of activation and root third. AP: AH Plus without activation; APU: AH Plus with ultrasonic activation; SP: Sealer Plus without activation; SPU: Sealer Plus with ultrasonic activation; BC: Sealer Plus BC without activation; BCU: Sealer Plus BC with ultrasonic activation.

Failure mode distribution (%) in each root third is displayed in Figure 1. The vast majority of specimens from all experimental groups had mixed failure. The relative frequency of mixed failures was equal or greater than 75%, 69%, 63% and 71% in the coronal, middle, apical third and overall, respectively.

**Table 2:** Push-out bond strength (MPa) according to sealer, type of activation and root third. Values were expressed in mean and standard deviation.

		Type of activation					
		No activation			Ultrasonic activation		
		Root canal sealer			Root canal sealer		
Root third	N	AP	SP	BC	APU	SPU	BCU
Coronal	16	2.20 ± 0.85^Aa^	1.70 ± 0.46^Aa^	1.70 ± 0.29^Aa^	2.11 ± 0.40^Aa^	2.16 ± 0.83^Aa^	1.94 ± 0.35^Aa^
Middle	16	1.80 ± 0.67^Aa^	2.08 ± 0.44^Aa^	1.67 ± 0.44^Aa^	2.12 ± 0.62^Aa^	2.06 ± 0.31^Aa^	1.75 ± 0.41^Aa^
Apical	16	1.59 ± 0.83^Ba^	2.29 ± 0.54^Aa^	1.61 ± 0.52^Bb^	1.86 ± 0.65^Aa^	2.53 ± 0.65^Aa^	2.34 ± 1.25^Aa^
Overall	48	1.86 ± 0.81^Aba^	2.02 ± 0.53^Aa^	1.66 ± 0.41^Bb^	2.03 ± 0.57^Aa^	2.25 ± 0.65^Aa^	2.01 ± 0.80^Aa^

AP: AH Plus; SP: Sealer Plus; BC: Sealer Plus BC; APU: AH Plus ultrasonically activated; SPU: Sealer Plus ultrasonically activated; BCU: Sealer Plus BC ultrasonically activated. Distinct uppercase letters indicate statistically significant difference between sealers (rows), while keeping type of activation and root third unchanged (P<0.05). Distinct lowercase letters indicate statistically significant difference between types of activation (rows), while keeping sealer and root third unchanged (P<0.05). No statistically significant difference was detected between root thirds (column) for any sealer and type of activation (P>0.05).

### Filling quality

**Figure 2:**
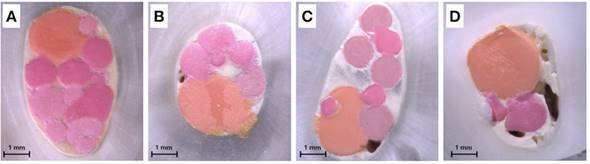
Representative stereomicroscopic images from slices of the middle third at 25× magnification. Score 1, SPU, Sealer Plus with ultrasonic activation (A); Score 2, BCU, Sealer Plus BC with ultrasonic activation (B); Score 3, BC, Sealer Plus BC without activation (C); Score 4, AP, AH Plus without activation (D).

The results of filling quality, represented by void scores, are expressed in Table 3 and Figure 2. In the groups without activation, BC showed higher void scores than SP in the apical third (P=0.014) and overall (P=0.008). In the ultrasonically activated groups, no significant difference was detected in any root segment (P>0.05). In the comparison between root thirds, AP, APU, and BC groups showed significantly higher scores in the apical third than in the coronal third (P=0.008; P=0.011; P=0.001, respectively). AP also presented higher scores in the middle third compared to the coronal third (P=0.021).

## Discussion

Recent investigations have evaluated the ultrasonic activation of endodontic sealers with different chemical compositions, showing mostly favorable results ^4^ ^,^ ^5^ ^,^ ^6^ ^,^ ^7^ ^,^ ^8^. This study assessed the bond strength and filling quality promoted by SP and BC, two relatively new materials, and the effect of ultrasonic activation on their properties. AP was used as the reference sealer for comparison, as described by several other authors ^5^ ^,^ ^6^ ^,^ ^7^ ^,^ ^9^. The null hypothesis was partially rejected because ultrasonic activation improved the bond strength of BC, but did not affect the filling quality of any sealer.

Regarding bond strength, comparison between groups without ultrasonic activation revealed no significant differences in the coronal and middle segments, but SP presented higher values than BC in the apical third and when data of all root thirds were pooled. Previous studies have explained the excellent bond strength performance of epoxy resin-based sealers by the ability to form a covalent bond to any exposed amino groups in dentin collagen when the epoxide ring opens ^18^. Interestingly, in the apical third, SP was also superior to AP. Both are epoxy resin-based sealers but have different compositions ^11^ and probably different viscosities. Possible differences in the proportion of resinous components (bisphenol-A and bisphenol-F epoxy resins) and the presence of calcium hydroxide in SP could explain these findings. Duarte and Moraes ^19^ have shown that calcium hydroxide addition leads to an improved sealing capacity of AP, as determined by dye infiltration. However, it is important to note that microleakage methodologies have been severely criticized, and no direct association can be established between apical seal and bond strength ^20^.

In this study, a significant improvement in the bond strength of BC was verified when the sealer was ultrasonically activated, which is in agreement with previous findings ^7^. This fact could be explained by the heat generated during the process, reducing sealer viscosity, combined with the transmission of acoustic streaming energy produced by the ultrasonic tip, forcing the sealer against the canal walls ^7^ ^,^ ^8^. There was also an increase in the bond strength of AP and SP, but it was not statistically significant. Despite using the ultrasonic insert with brushing movements, the wide diameter of the root canals of bovine teeth may be responsible for the lack of significant difference between activated and

**Table 3:** Void scores according to sealer, type of activation and root third.

			Root third																				
			Coronal					Middle					Apical					Overall					
			Scores				Median	Scores				Median	Scores				Median	N	Scores				Median
Sealer	Type of activation	N	1	2	3	4		1	2	3	4		1	2	3	4			1	2	3	4	
AH Plus	NA	16	9	5	2	0	1^Ab^	3	6	3	4	2^Aa^	4	3	8	1	3^ABab^	48	16	14	13	5	2^AB^
	UA	16	13	1	2	0	1^Ab^	8	0	6	2	2^Aab^	5	0	10	1	3^Aa^	48	26	1	18	3	1^A^
Sealer Plus	NA	16	9	4	3	0	1^Aa^	8	3	4	1	1.5^Aa^	7	2	7	0	2^Ba^	48	24	9	14	1	1.5^B^
	UA	16	8	5	3	0	1.5^Aa^	7	6	3	0	2^Aa^	6	3	6	1	2^Aa^	48	21	14	12	1	2^A^
Sealer Plus BC	NA	16	11	3	2	0	1^Ab^	3	2	10	1	3^Aab^	0	3	10	3	3^Aa^	48	14	8	22	4	3^A^
	UA	16	7	7	2	0	2^Aa^	7	2	6	1	2^Aa^	5	2	7	2	3^Aa^	48	19	11	15	3	2^A^

NA: No activation; UA: Ultrasonic activation. Distinct uppercase letters indicate statistically significant difference between sealers (column), while keeping type of activation and root third unchanged (P<0.05). Distinct lowercase letters indicate statistically significant difference between root thirds (rows), while keeping sealer and type of activation unchanged (P<0.05). No statistically significant difference was detected between types of activation for any sealer and root third (P<0.05).

non-activated resinous sealers. The possibility of a substantial improvement in narrower canals cannot be discarded. It is noteworthy that Wiesse et al. ^7^ found better results when AP was ultrasonically activated, but the authors used root canals of human teeth with more restricted apical sizes.

The absence of significant differences between root thirds regarding bond strength, as observed here, has already been reported ^16^. Endodontic sealers were introduced into the canals by a Lentulo spiral, which allows a more homogeneous distribution of the sealer up to the apex ^21^. Moreover, according to Dash et al. ^22^, the sealer achieves greater penetrability into the dentinal tubules when applied by this instrument. In this context, the material can show adequate adhesion to dentin even at the apical root third.

The mixed failure mode, where adhesive and cohesive failures coexist, was the most common failure pattern induced by the push-out test, representing more than 60% of the specimens in all experimental groups and root thirds. These findings are in accordance with past studies ^7^ ^,^ ^17^. We must consider that such failures may have occurred because the force applicator tip was always the same, which may have affected the results, as it infringes force only on the central part of the filling material. There is no consensus in the endodontic literature concerning the failure mode observed with bioceramic and resinous sealers, probably because of differences in the methodological setting ^7^ ^,^ ^16^ ^,^ ^17^ ^,^ ^18^.

One of the ways to analyze root canal filling quality is through its visual observation. A recent study compared the filling quality promoted by different endodontic sealers using micro-computed tomography (micro-CT), followed by stereomicroscopic observation of root sections ^6^. No significant difference was found between groups when evaluated by micro-CT, whereas in the stereomicroscopic analysis, a pre-mixed bioceramic sealer (Endoseal MTA; Maruchi, Wonju, Korea) showed a higher number of voids than AP. Those authors speculated that micro-CT might be less sensitive than the sectioning method in terms of void detection since sealers are considerably radiopaque, which may impair the micro-CT detection of small voids within the bulk of the root filling.

In the present investigation, the second slice of each root third was examined under a stereomicroscope and scored, as described in the study mentioned above ^6^. In groups without ultrasonic activation, BC presented significantly higher void scores than SP in the apical third, and when data of root thirds were polled. On the other hand, BC produced similar void scores compared with AP, regardless of ultrasonic activation. A previous study also found that pre-mixed bioceramic sealers and AP promote the same filling quality ^23^.

Unlike bond strength results, filling quality was not improved by ultrasonic activation in this study. Similarly, Guimarães et al. ^5^ evaluated four epoxy resin-based sealers and did not detect differences in void percentage when they were ultrasonically activated, despite observing greater penetration of sealers into the dentinal tubules. Kim et al. ^6^ found lower void scores when ultrasound was applied, but they used a gutta-percha cone-mediated ultrasonic activation, in which the ultrasonic tip did not contact the sealer, but a cotton plier that held the gutta-percha cone.

Thus, ultrasonic activation of sealers did not seem to influence the presence of voids, which probably is more related to the inability of the lateral condensation technique to allow a dense and homogeneous obturation ^5^. In this study, ultrasound only acted in the adaptation of the sealer to the canal walls, before starting lateral condensation procedures. Better outcomes could be obtained if ultrasound had been used to activate the spreader while inserting accessory gutta-percha points into the canal, as described by other authors ^1^. Furthermore, it can the hypothesized that increasing ultrasonic power would improve filling quality. However, it would also increase the risk of fracture of the ultrasonic insert. The power recommended for this purpose in previous studies ranges from 10% to 50% ^5^ ^,^ ^7^ ^,^ ^22^.

When root thirds were compared, there was a tendency to lower void scores in the coronal third, increasing towards the apex. Significant differences were observed for AP, APU, and BC. This finding may cause some concern since voids and gaps in the apical third may be connected with dentinal tubules, accessory canals, or other ramifications that may harbor microorganisms. It has been shown that a persistent infection in this apical segment is the main cause of endodontic treatment failure ^24^.

The use of bovine teeth with round and wide root canals may be pointed as a limitation of the present study. The root filling could be much more challenging when oval canals, isthmuses and other ramifications are involved. These conditions should be investigated in further studies, and the effect of ultrasonic activation could be more pronounced ^4^. 

The push-out test is a widely used and well-accepted method to evaluate the bond strength of root filling materials in root canals, but it has inherent limitations and variations in the test may influence the results. According to Pane et al. ^25^, the punch diameter should be 70-90% of the canal size. The push-out strength can be underestimated when the punch diameter is 50-60% of the canal size. In this study, a standardized 0.8-mm tip was applied in the center of the filling material, without contacting the canal walls. The apical preparation was performed up to a size 100 K-file, so the punch diameter seems appropriate in the apical root third but may have led to an underestimation in the middle and cervical sections.

In conclusion, within the limits of this study, ultrasonic activation was effective in increasing the bond strength of the calcium silicate-based sealer (BC) but did not improve its filling quality. For the epoxy resin-based sealers (AP and SP), these properties were not affected by the use of ultrasound.
